# Validation of a highly sensitive HaloTag-based assay to evaluate the potency of a novel class of allosteric β-Galactosidase correctors

**DOI:** 10.1371/journal.pone.0294437

**Published:** 2023-11-29

**Authors:** Mikhail Rudinskiy, Maria Pons-Vizcarra, Tatiana Soldà, Ilaria Fregno, Timothy Jan Bergmann, Ana Ruano, Aida Delgado, Sara Morales, Xavier Barril, Manolo Bellotto, Elena Cubero, Ana María García-Collazo, Natalia Pérez-Carmona, Maurizio Molinari

**Affiliations:** 1 Università Della Svizzera Italiana, Lugano, Switzerland; 2 Department of Biology, Swiss Federal Institute of Technology, Zurich, Switzerland; 3 Institute for Research in Biomedicine, Bellinzona, Switzerland; 4 Gain Therapeutics Sucursal en España, Parc Científic de Barcelona, Barcelona, Spain; 5 Facultat de Farmacia, IBUB & IQTC, Universitat de Barcelona, Barcelona, Spain; 6 Catalan Institution for Research and Advanced Studies (ICREA), Barcelona, Spain; 7 GT Gain Therapeutics SA, Lugano, Switzerland; 8 School of Life Sciences, École Polytechnique Fédérale de Lausanne, Lausanne, Switzerland; University of Ferrara, ITALY

## Abstract

Site-directed Enzyme Enhancement Therapy (SEE-Tx®) technology is a disease-agnostic drug discovery tool that can be applied to any protein target of interest with a known three-dimensional structure. We used this proprietary technology to identify and characterize the therapeutic potential of structurally targeted allosteric regulators (STARs) of the lysosomal hydrolase β-galactosidase (β-Gal), which is deficient due to gene mutations in galactosidase beta 1 (GLB1)-related lysosomal storage disorders (LSDs). The biochemical HaloTag cleavage assay was used to monitor the delivery of wildtype (WT) β-Gal and four disease-related β-Gal variants (p.Ile51Thr, p.Arg59His, p.Arg201Cys and p.Trp273Leu) in the presence and absence of two identified STAR compounds. In addition, the ability of STARs to reduce toxic substrate was assessed in a canine fibroblast cell model. In contrast to the competitive pharmacological chaperone N-nonyl-deoxygalactonojirimycin (NN-DGJ), the two identified STAR compounds stabilized and substantially enhanced the lysosomal transport of wildtype enzyme and disease-causing β-Gal variants. In addition, the two STAR compounds reduced the intracellular accumulation of exogenous GM1 ganglioside, an effect not observed with the competitive chaperone NN-DGJ. This proof-of-concept study demonstrates that the SEE-Tx® platform is a rapid and cost-effective drug discovery tool for identifying STARs for the treatment of LSDs. In addition, the HaloTag assay developed in our lab has proved valuable in investigating the effect of STARs in promoting enzyme transport and lysosomal delivery. Automatization and upscaling of this assay would be beneficial for screening STARs as part of the drug discovery process.

## Introduction

Only correctly folded proteins are released from the endoplasmic reticulum (ER) and transported to their intra- or extracellular site of activity [1]. Misfolded polypeptides are eventually dislocated across the ER membrane for ER-associated degradation or segregated in specialized ER subdomains that are cleared by ER-to-lysosome-associated degradation [2, 3]. Mutations in the polypeptide’s sequence substantially reduce the folding efficiency and may result in loss-of-function phenotypes or related diseases [4]. Autosomal recessive mutations in the galactosidase beta 1 (*GLB1*) gene encoding the lysosomal hydrolase β-galactosidase (β-Gal) manifest in phenotypically distinct GLB1-related lysosomal storage disorders (LSD) [5, 6]. For example, the p.Ile51Thr mutation is common among adult Japanese patients affected with late-onset GM1-gangliosidosis [7], p.Arg59His causes severe infantile GM1-gangliosidosis with cardiac involvement [8, 9], p.Arg201Cys is associated with late infantile/juvenile type 2 GM1-gangliosidosis [10, 11], and p.Trp273Leu has been linked with juvenile Morquio B disease [12]. In patients with the mutations mentioned above, the intracellular β-Gal activity is partially or totally lost, resulting in the toxic accumulation of its two main substrates, GM1-ganglioside and keratan sulfate [13]. Most *GLB1* mutations, however, do not abolish the enzymatic function of β-Gal but result in minor structural defects caused by the point mutations, which delay the attainment of the native structure [7, 10, 13–17]. This delay alerts the protein quality control machinery that retains the aberrant protein in the ER and eventually selects it for degradation.

Pharmacological chaperones have been shown to improve the folding and/or stability of target proteins such as β-Gal, increasing the overall quantity of active enzyme reaching its target location [18]. Most pharmacological chaperones are competitive inhibitors that occupy the enzyme’s active site; notably, their therapeutic effect is limited by their inhibitory activity, e.g., N-nonyl-deoxygalactonojirimycin (NN-DGJ) [19]. Non-competitive, allosteric pharmacological chaperones, therefore, represent a promising therapeutic alternative to competitive inhibitors in stabilizing the mutant proteins and improving their delivery to their site of action (e.g., delivery of β-Gal to lysosomes) without incurring inhibitory effects [20–27].

Recently, we developed HaloTag-based assays to quantitatively monitor events related to autophagy, i.e., to follow the delivery of ER portions to endolysosomes for clearance, which occurs via *micro*-ER-phagy or LC3-mediated vesicular delivery [28–33]. HaloTag is a modified bacterial haloalkane dehalogenase whose active site has been engineered to covalently bind cell-permeable chloro- and bromo-alkanes modified with fluorescent ligands such as tetramethylrhodamine (TMR) [34, 35]. Like for other conventionally used protein tags (e.g., HA, V5, FLAG, His), commercial antibodies to HaloTag are used to monitor the fate of the protein-of-interest displaying the selected tag at the N- or C-terminus in imaging and biochemical analyses [35]. The advantage of the HaloTag system compared to other protein tags is that it can be fluorescently labeled on demand by adding small, cell-permeable, fluorescent HaloTag ligands in the cell culture media [35]. Thus, a chimeric protein of interest can be seen in light and electron microscopy directly, with no need for fluorescent or gold-labeled secondary antibodies [35]. Furthermore, when the chimeric protein-of-interest is delivered to lysosomal compartments, the HaloTag is cleaved to generate acid- and protease-resistant, fluorescent HaloTag fragments that can directly be visualized to offer a very sensitive and quantitative measure of lysosomal delivery [32, 33, 36]. In contrast to the large polypeptide size of HaloTag (33 kDa), most other protein tags are short peptides of 6–12 residues that do not perturb protein biogenesis and intracellular location [37].

In this study, the innovative drug discovery platform, Site-directed Enzyme Enhancement Therapy (SEE-Tx®) [38], was used to virtually screen and identify new allosteric druggable binding sites and discover structurally targeted allosteric regulators (STARs) of β-Gal with therapeutic potential. In addition, to determine the therapeutic potential and mechanism of action of the putative STARs, we monitored lysosomal delivery of wildtype (WT) β-Gal and four disease-related β-Gal variants p.Ile51Thr, p.Arg59His, p.Arg201Cys and p.Trp273Leu using the sensitive, quantitative, versatile protein-tag system (HaloTag) [35].

## Materials and methods

### Identification of STARs

The innovative proprietary drug discovery platform SEE-Tx^®^ was applied to identify druggable binding sites different from the active site using the published 3D structure of human β-Gal obtained by X-ray crystallography and refined to 1.8 Å resolution [PDB ID: 3THC] [39]. The druggability of the putative allosteric binding site identified was confirmed with a physics-based method consisting of molecular dynamics simulations of the protein in organic-aqueous solvent mixtures [40]. The same method was used to identify key interactions (binding hot spots), which were used as pharmacophoric restraints to guide docking [41–43]. A virtual collection of 4,840,400 unique chemical compounds commercially available from the chemical providers Asinex, Enamine, Key Organics, Life Chemicals and Specs was evaluated with the docking program *rDock* using the standard scoring function, pharmacophoric restraints, and a high-throughput protocol [44]. The best scoring compounds (n = 9133) were visually assessed to determine their suitability as a potential small molecule drug. A subset of virtual hits (n = 144) was selected based on chemical complementarity to the proposed β-Gal protein based on docking-generated binding mode and chemical diversity considerations. The virtual hits binding to β-Gal was experimentally validated by differential scanning fluorimetry (DSF) assay [45], as previously described [46]. The validated hits were then subjected to a hit-to-lead optimization process.

**Fig 1:**
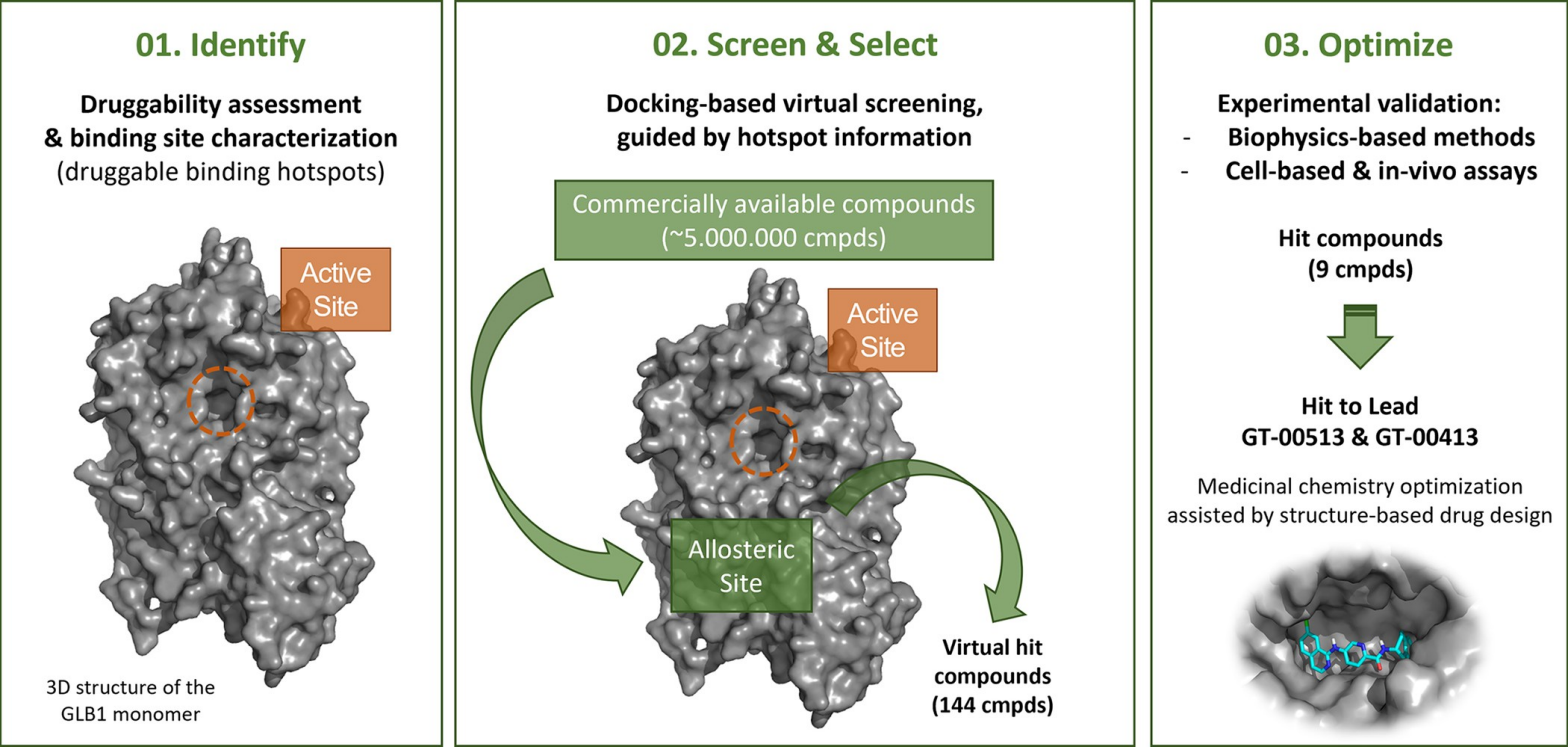
Self-explanatory graphic of the SEE-Tx® methodology used to discover non-competitive, pharmacological allosteric regulators for β-Galactosidase.

An overview of the SEE-Tx® platform methodology is shown in Fig 1.

### Surface Plasmon Resonance (SPR) binding assay

All SPR experiments were performed at 20°C on Biacore T200 (GE Healthcare, Uppsala, Sweden). The SPR technique detects changes in the refractive index on the sensor’s surface due to mass changes. These changes measure the biomolecular interaction between the purified β-Gal protein and the validated hit compounds.

#### Protein immobilization

Human full-length β-Gal protein (recombinant human β-Galactosidase) from a Chinese hamster ovary (CHO) cell line with a C-terminal 6-his tag, Met1-Val677 in 25mM Tris and 150mM NaCl, pH 7.5 (Bio-Techne, Minneapolis, Minnesota, U.S.) was immobilized on the SPR CM5 sensor (29149603, GE Healthcare) by standard amino coupling using relatively high protein concentration of 100 μg/mL^-1^ in 10mM Na acetate buffer (pH 4.0) to reach protein density levels of ~10000 Response Units (RU) (10000 RU channel 2 & 12700 RU channel 3). Running buffer (PBS, pH 7.0) was used for protein immobilization. Empty, activated, and deactivated parallel channels on the same SPR sensor were used as reference channels. Protein binding activity was probed with a reference compound, NN-DGJ (K_D_ of 1.1 μM) in a dose-dependent manner.

#### Binding studies with small molecule β-Gal ligands

SPR binding studies were performed at pH 7.0 and pH 5.0. Two SPR running buffers (supplemented with dimethylsulfoxide [DMSO]) were used for these studies: phosphate-buffered saline (PBS), 5% (v/v) DMSO (pH 7.0) and 20mM sodium phosphate, 2.7mM KCl, 137mM NaCl, 5% (v/v) DMSO (pH 5.0). Small molecule ligands were diluted with the appropriate running buffer from 10mM DMSO stock solutions to obtain a concentration series of 9 points with a 2-fold serial dilution. Compounds were titrated in duplicates over the β-Gal protein surface up to 12.5 μM for GT-00513 at both pHs and GT-00413 at pH 5.0, and up to 60 μM for GT-00413 at pH 7.0.

#### SPR data analysis

Raw SPR signals monitored on the active channel (with the immobilized target protein) were subtracted from signals monitored on the reference channel (empty sensor surface) and further subtracted from the signal monitored for the running buffer (double referenced), and finally corrected for DMSO signal mismatch between sample and running buffer. To extract binding affinity values, the plotted SPR data were further fitted with the four-parameter logarithmic dose-response equation (GraphPad Prism, Version 9.4.0) without constraint.

### Cell culture, transfection, and use of compounds

Mouse embryonic fibroblasts (MEF) and Human Embryonic Kidney 293 (HEK293) cells were cultivated in Dulbecco’s Modified Eagle Medium (DMEM) with high glucose (GlutaMAX™, Gibco) supplemented with 10% Fetal calf serum (FCS, Gibco) at 37° C and 5% CO_2_. Transient transfections were performed in DMEM 10% FCS and supplemented with non-essential amino acids (NEAA, Gibco) using JetPrime (Polyplus) following the manufacturer’s protocol. Lysosomal acidification (and degradation) was inhibited using 50nM Bafilomycin A1 (BafA1, Millipore) in DMEM 10% FCS for 15h prior to fixation or cell lysis. Novel allosteric pharmacological chaperones synthesized by GAIN Therapeutics (GT) and known competitive chaperone (NN-DGJ) were used at 25μM in DMEM, 10% FCS supplemented with NEAA over a period of four days. WT β-Gal (ref. SV-2025-V04, STEMNOVATE) and p.Arg60His/p.Arg60His-β-Gal canine fibroblasts (ref. GM11473, Coriell) were cultured in DMEM (ref. 11574486, Gibco) with 10% FCS (ref. 10500, Gibco) and 1% penicillin/streptomycin (P/S) (10,000 μg/mL) (ref. 15140122, Gibco) in 96 well plates (ref. 89626, IBIDI) at 37°C and 5% CO_2_. At 48 h after seeding, exogenous GM1 bovine ganglioside (ref. G7641, Sigma Aldrich) was added at a final concentration of 0.1 mg/ml. Two days after that, compounds (or DMSO as control) were added at the indicated concentration for four subsequent days. Canine fibroblast cultures were fixed with 4% paraformaldehyde (ref. 15710, Electron Microscopy Sciences) in PBS for 15 min at room temperature (RT).

### Plasmids and cloning

β-Gal-HA (human influenza hemagglutinin) plasmid in pcDNA3.1(+) backbone was synthesized by GenScript. β-Gal mutants of interest were generated by site-directed mutagenesis polymerase chain reaction (PCR) using the QuikChange II site-directed mutagenesis kit (Agilent) following the manufacturer’s protocol. Primers (S1 Table) were purchased from MicroSynth AG. Exchange of the HA and HaloTag tags between the different plasmids was obtained by restriction digestion of NotI and XhoI sites flanking the tags. Ligation was performed with T4 DNA ligase (NEB) with a 3:1 (insert: vector ratio) following the manufacturer’s protocol. Plasmids were amplified and isolated from JM109 bacteria (Promega) using GenElute™ HP plasmid MidiPrep kit (Sigma).

### Immunocytochemistry and microscopy imaging

MEF cells were plated on alcian blue-coated coverslips in a 12-well plate, allowed to set for 10–17 h, and transfected as mentioned above. β-Gal constructs were expressed for 24 h. Media was replaced 15 h before fixation, and DMEM 10% FCS supplemented with 100nM HaloTag TMR ligand (Promega), and 50nM BafA1 (Millipore) or DMSO (Sigma) was added. Cells were fixed in 3.7% PFA (Sigma) in DPBS (Gibco) for 20 min at RT and washed three times with DPBS (Gibco). Cells were permeabilized for 20 min at RT with PS solution (0.05% saponin, Sigma), 15 mM Glycine (AppliChem), 10 mM HEPES (Gibco), 10% goat serum (in DPBS, Gibco). Coverslips were then placed onto a drop of anti-LAMP1 antibody (Hybridoma bank) (S2 Table) or a mixture of anti-LAMP1 and anti-HA (Sigma) (S2 Table) antibodies diluted in PS solution and incubated in a wet chamber at RT for 90 min. Samples were washed three times in PS solution, placed back onto a drop of anti-rat Alexa647-conjugated secondary antibody (Thermo Fisher Scientific) (S2 Table) or a mixture of anti-rat Alexa647-conjugated and anti-rabbit Alexa568-conjugated secondary antibodies (Thermo Fisher Scientific) (S2 Table) diluted in PS solution for 45 min at RT in a dark wet chamber. After washing three times in PS solution and rinsing in ddH_2_O, coverslips were mounted on a microscopy slide using VectaShield supplemented with 4′,6-diamidino-2-phenylindole (DAPI, Vector laboratories Inc.) and fixed with nail polish. Confocal images were acquired on a Leica TCS SP5 microscope with a Leica HCX PL APO lambda blue 63.0 × 1.40 OIL UV objective with pinhole 1 AU. Excitation was performed with 561 nm (HaloTag/TMR) and 633 nm (LAMP1) lasers and fluorescence light was collected in 566–628 and 640–702 nm ranges, respectively. Image analysis and quantification were performed with FIJI [50] and LysoQuant [51]. Image processing was performed in Photoshop (Adobe), while plotting of data and statistical analysis was performed with GraphPad Prism 9 (GraphPad Software Inc.).

Canine fibroblasts were permeabilized for 15 min at RT with PBS containing 0.3% Triton X-100 (ref. T8787, Sigma-Aldrich). Cytoplasm was labeled using HCS CellMask (Thermofisher) (S2 Table) for 15 min at RT. Subsequently, fibroblasts were blocked with PBS containing 0.5% Bovine Serum Albumin (BSA, ref. A9647, Sigma-Aldrich) and 10% normal donkey serum (ref. S30, EMD Millipore) for 1 h at RT. All antibodies were diluted in PBS containing 0.5% BSA; incubation with primary antibody against Ganglioside GM1 (Abcam) (S2 Table) was undertaken overnight at 4°C. Alexa Fluor® 488 Donkey anti-Rabbit IgG (Invitrogen) (S2 Table) was used as a secondary antibody for 1 h at RT. Nuclei were counterstained using DAPI (ThermoFisher Scientific) in PBS for 10 min at RT. Fibroblasts were washed and stored at 4°C in PBS until imaging. The stained cells were imaged using a LIPSI Nikon microscope. Images were taken using a 40x water immersion objective (NA = 1.15).

Twenty-five images in each channel were acquired per well, with a 10% overlap (5x5 square) and a frame size of 2048x2048 pixels. The analysis of the images was primarily executed using the NIS software analysis tool (NIS-Elements AR 5.21). First, stitching was performed with the 25 images to obtain a single image per well with three channels. From the single image, Cell Mask staining was used to delimitate the cell area and DAPI to delimitate nuclei area. Green dots (GM1 ganglioside) intensity, size and number were determined inside the cytoplasm, excluding the nuclei area. A ratio between the area of GM1 ganglioside and the total cell area was calculated to consider the differences in cell size. In addition, only cells with nuclei were included in the analysis.

### Cell lysis, SDS-PAGE, Western blot and HaloTag® cleavage assay

HEK293 cells were plated on poly-lysine (Sigma)-coated 24-well plates, allowed to set for 8 h and transfected as described above. At 15–17 h after transfection, media was replaced with DMEM 10% FCS supplemented with NEAA (Gibco) containing compounds (GT-00513 or GT-00413) or DMSO. Fifteen hours before lysis, TMR and BafA1 were added directly to the culture media at a final concentration of 100nM and 50nM, respectively. After a total of four days of treatment with the GT compounds (GT-00513 or GT-00413), plates were put on ice, cells washed with ice-cold DPBS supplemented with 20 mM N-ethylmaleimide (NEM) and lysed in 100 μl RIPA lysis buffer (1% Triton-X-100, 0.1% SDS, 0.5% Na-deoxycholate in HBS buffer pH 7.4) containing 20mM NEM and protease inhibitors (1 mM PMSF and CLAP mix). After 20 min on ice, samples were centrifuged at 10600 *g* at 4°C for 10 min, and the post-nuclear supernatant (PNS) was collected. Sodium dodecyl sulfate-polyacrylamide gel electrophoresis (SDS-PAGE) was performed with 10 μL of PNS denatured with sample buffer containing 100nM DTT at 95°C for 5 min and loaded on a 10% acrylamide gel. TMR signal was detected on a Typhoon™ FLA 9500 fluorescent scanner (GE Healthcare). TMR bands were quantified using the ImageQuantTL software (Molecular Dynamics, GE Healthcare). For quantification of total protein, gels were further stained 20 min at RT with 0.25% Brilliant blue R 250 (Sigma) solution (50% MetOH, 10% acetic acid) and thoroughly destained in 20% MeOH, 7.5% acetic acid solution, before acquiring sample image on a Fusion FX7 system (Vilber) with transilluminator.

For detection of HaloTag by immunoblot (IB), protein was transferred to the polyvinylidene fluoride (PVDF) membrane using a TransBlot Turbo device (BioRad). PVDF membrane was rinsed in MetOH, then washed in Tris-buffered saline, 0.1% Tween 20 (TBS-T), blocked 10 min at RT with 10% blocking milk (BioRad) in TBS-T, washed quickly in TBS-T and incubated with anti-HaloTag antibody (Promega) (S2 Table) diluted in TBS-T overnight at 4°C. After washing in TBS-T, the membrane was incubated with anti-mouse HRP-conjugated secondary antibody (Southern Biotech) (S2 Table) diluted in TBS-T for 45 min at RT. Detection was performed using WesternBright™ Quantum (Advansta) following the manufacturer’s protocol and imaged on a Fusion FX7 chemiluminescence detection system (Vilber).

### Statistical analyses

Statistical comparisons and graphical plots were created using GraphPad Prism 9 (GraphPad Software Inc.). Data are expressed as mean ± standard deviation (SD). In this study, an ordinary one-way ANOVA Dunnett’s multiple comparisons test was used to assess statistical significance (for Figs 4B, 4C, 5B, 5C, 5E, 6B, 6D, 6F, 6H, 6J, 7B and 7C). An adjusted P value < 0.05 (for one-way ANOVA with Dunnett’s multiple comparison test) was considered statistically significant; significance is denoted: ns P>0.05, * P<0.05, ** P<0.01, *** P<0.001, **** P<0.0001. All experimental replicates represent biological replicates.

## Results

### Allosteric binding site and hit identification

Using the 3D structure of GLB1 protein [39] and supercomputing technology [40–43], SEE-Tx® platform revealed a novel druggable allosteric site different from the active site. The same method was used to identify key interactions sites (binding hotspots), which were converted to pharmacophoric restraints to guide docking [44]. Out of 4,820,400 input molecules, 9133 fulfilled the pharmacophoric restraints and obtained a docking intermolecular score lower than -18.0 units. These compounds were ranked by normalised score (intermolecular docking score divided by the number of non-hydrogen atoms) and clustered by similarity based on MACCS fingerprints. Visual inspection of a diverse set of top-ranked compounds led to the selection of 144 molecules that were purchased and tested experimentally in the DSF assay [46], resulting in several hit compounds (n = 9) that were used as starting points for a hit-to-lead medicinal chemistry program.

### GT-00413 and GT-00513, structurally targeted allosteric regulators (STARs)

**Fig 2:**
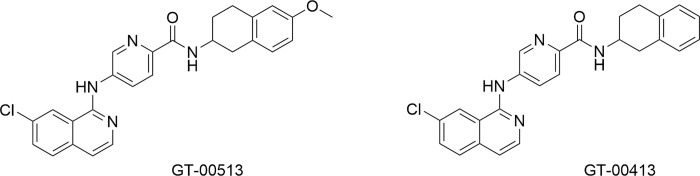
Chemical structure of GT-00513 and GT-00413.

The hit compounds identified applying our proprietary SEE-Tx® technology were used as the basis for a medicinal chemistry program that after several rounds of optimization, yielded the lead compounds GT-00513 and GT-00413 [47]. Their chemical structure is depicted in Fig 2.

In biochemical assays performed in purified human β-Gal protein and lysates from human WT fibroblasts and using resorufin beta-D-galactopyranoside as substrate, the pharmacological chaperone NN-DGJ inhibited the endogenous β-Gal activity in a dose-dependent manner as expected for a competitive inhibitor. In sharp contrast, GT-00513 and GT-00413 did not display inhibition of the β-Gal activity at all concentrations tested, confirming their action as non-competitive pharmacological chaperones (S1 File).

Furthermore, the compounds present drug-like ADME properties and were shown to be brain penetrant and orally bioavailable.

### Confirmation of STARS binding to β-Gal by surface plasmon resonance (SPR)

**Fig 3:**
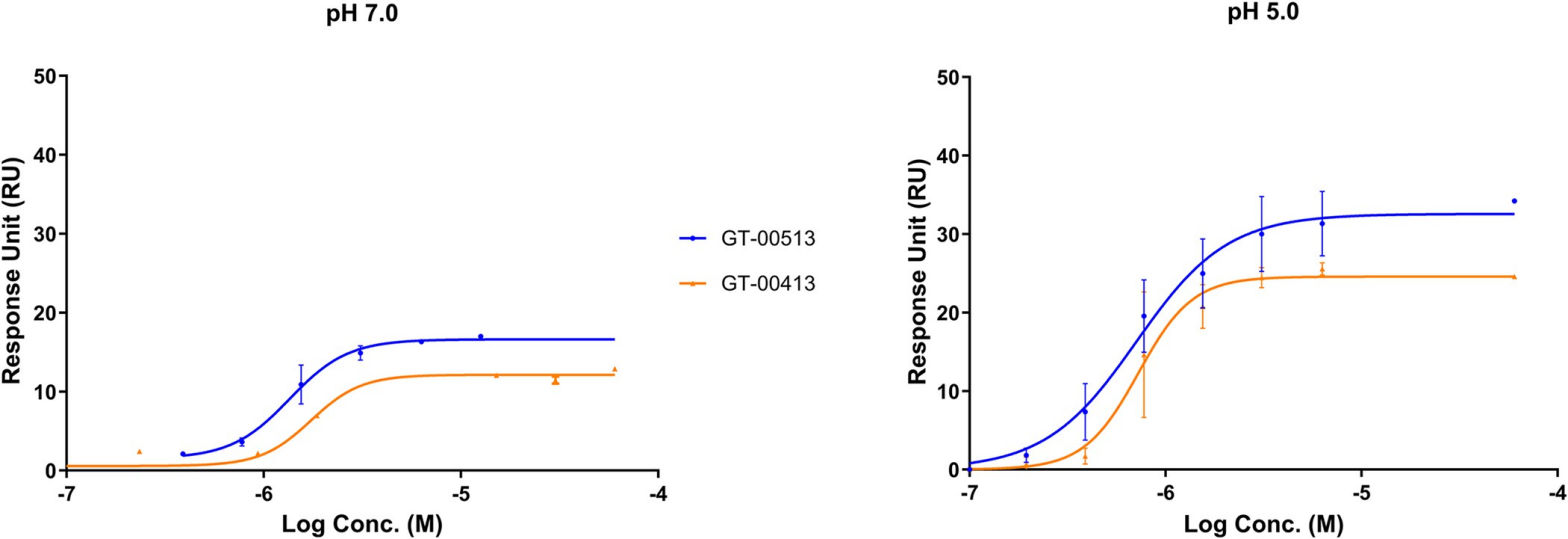
Surface plasmon resonance (SPR) dose-response for GT compounds binding to immobilized β-galactosidase monitored at neutral (7.0) and acidic (5.0) pH.

Analysis of the direct binding of GT-00513 and GT-00413 to β-Gal was performed using surface plasmon resonance (SPR). SPR enables the study of molecular interactions [48, 49] and calculates the dissociation constant (K_D_) of ligand binding. Direct binding to recombinant human β-Gal WT protein of STARs was confirmed in a dose-response manner at neutral pH 7.0 and at acidic pH 5.0 (Fig 3). The affinity of binding (K_D_) for GT-00513 and GT-00413 at both pHs are shown in Table 1.

**Table 1:** SPR direct binding affinities (K_D_) measured at pH 7.0 and 5.0 for compounds GT-00513 and GT-00413.

Compound	Dose response (pH 7.0)	K_D_ (μM)	Dose response (pH 5.0)	K_D_ (μM)
NN-DGJ (control)	Yes	0.37–1.12	No	N.D.
GT-00513	Yes	0.49–0.83	Yes	0.23–0.50
GT-00413	Yes	0.64–2.21	Yes	0.54–0.66

Abbreviations: K_D_, dissociation constant; NN-DGJ, N-nonyl-deoxygalactonojirimycin; N.D. Not determined.

Notably, the K_D_s of lead compounds are in the same order of magnitude as measured for the control, N-nonyl-deoxygalactonojirimycin (NN-DGJ), at pH 7.0. Also, at pH 5.0, the novel compounds show a similar K_D_ compared to neutral pH. In contrast, at pH 5.0, the control inhibitor NN-DGJ does not bind at the optimal acidic pH conditions for GT compounds.

### Lysosomal delivery of β-Gal WT and variants

To assess the usability of the HaloTag system in our study, i.e., straightforward, quantitative assessment of pharmacological chaperoning of β-Gal variants, we first compared the fate of HA- versus HaloTag-tagged versions of the four β-Gal variants p.Ile51Thr, p.Arg59His, p.Arg201Cys and p.Trp273Leu.

**Fig 4:**
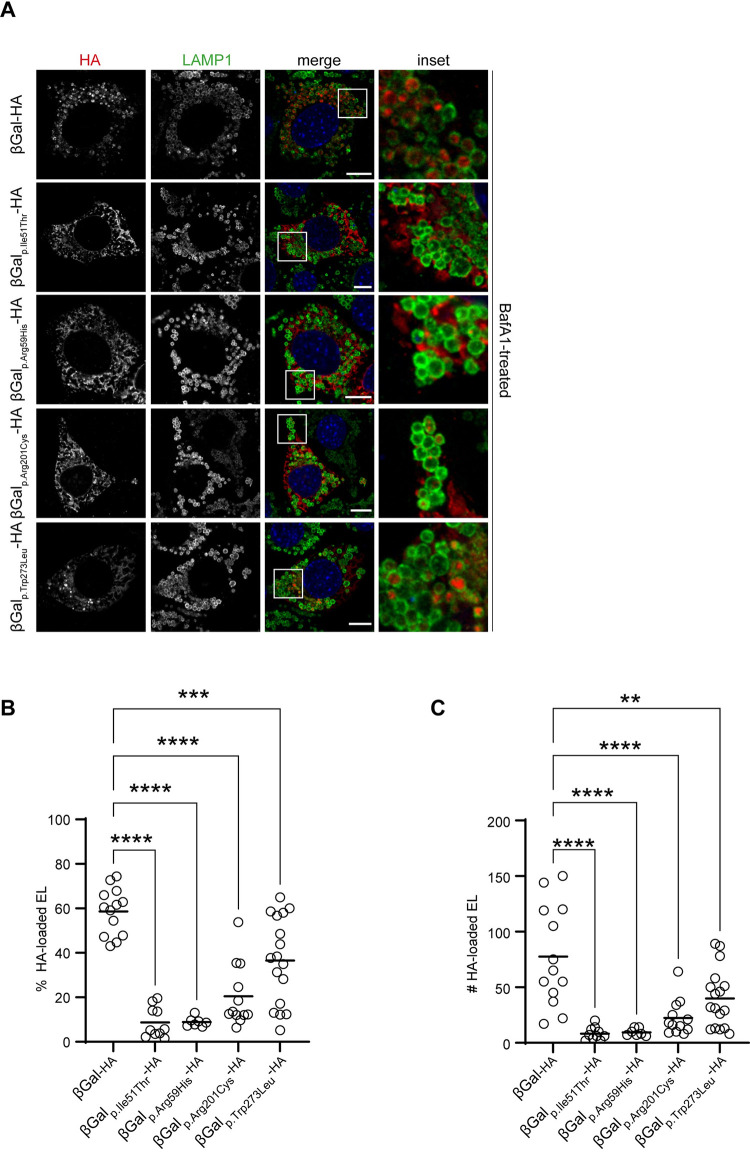
Lysosomal delivery of β-Gal-HA variants. **A.** Lysosomal delivery of β-Gal-HA variants. Representative confocal IF images of MEF cells expressing β-Gal-HA variants for 24 h and exposed 15 h to 50nM BafA1. β-Gal-HA immunostained with anti-HA antibody (red) and endolysosomes with anti-LAMP1 antibody (green). Nuclei are stained with DAPI (blue). Scale bar 10 μm (merge), inset scale: 4x the magnification of the merge image. **B**, Quantification of **A** as percentage of lysosomes filled with HA. Images analyzed with LysoQuant. Statistical analysis: One-way ANOVA followed by Dunnett’s multiple comparison test, ***P<0.001, ****P<0.0001; n = 13, 10, 7, 12 and 17 cells, respectively; n = 1 biological replicate. Mean bar is shown. **C.** Quantification of **A** as absolute number of lysosomes filled with HA per cell. Images analyzed with LysoQuant. Statistical analysis: One-way ANOVA followed by Dunnett’s multiple comparison test, ^ns^P>0.05, **P<0.01, ****P<0.0001. Mean bar is shown. **D.** Schematic representation of disease-severity. Abbreviations: β-Gal, beta-galactosidase; DAPI, 4′,6-diamidino-2-phenylindole; EL, endolysosomes; IF, immunofluorescence; MEF, mouse embryonic fibroblasts; BafA1, Bafilomycin A1, HA, human influenza hemagglutinin.

The MEFs seeded on glass coverslips were transiently transfected with plasmids encoding the HA-tagged versions of WT β-Gal (β-Gal-HA) and the four disease-causing variants, as described in the Methods section (Fig 4A–4C). Ten hours post-transfection, 50nM of Bafilomycin A1 (BafA1) was added to the cell medium to inhibit lysosomal activity [54], stabilizing the β-Gal polypeptides delivered within the target organelles. Then, cells were processed for confocal laser scanning microscopy (CLSM) as described in the Methods section and immunostained with antibodies for the lysosomal marker LAMP1 (green) and for the HA-tag (red) to evaluate the intra-lysosomal delivery of the β-Gal variants (Fig 4A). Analyses of the micrographs (Fig 4A) and their quantification with LysoQuant (Fig 4B and 4C) [29, 32, 50, 51] reveal that disease-causing mutations in the β-Gal sequence substantially reduce the percentage (Fig 4B) and the number (Fig 4C) of cellular LAMP1-positive lysosomes containing the ectopically expressed lysosomal enzymes. Notably, the severity of the phenotype is also stronger for the p.Ile51Thr and p.Arg59His mutations and milder for the p.Trp273Leu mutation versus WT (Fig 4D).

**Fig 5:**
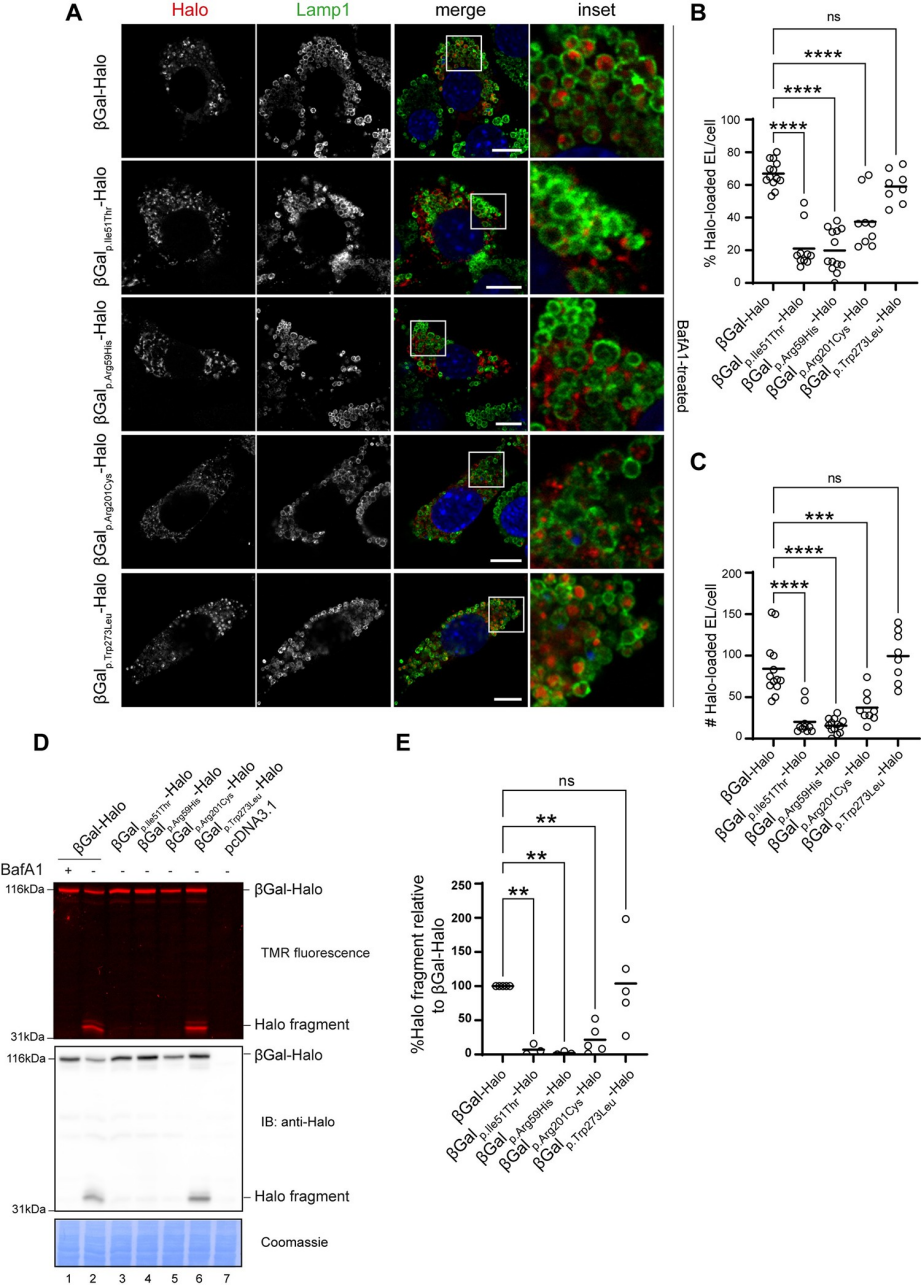
Lysosomal delivery and maturation of β-Gal-HaloTag variants. **A.** Lysosomal delivery of β-Gal-HaloTag variants. Representative confocal IF images of MEF cells expressing β-Gal-HaloTag variants for 24 h and exposed for 15 h to 50nM BafA1. β-Gal-HaloTag stained with 100nM TMR HaloTag ligand (red) and lysosomes with anti-LAMP1 antibody (green). Nuclei are stained with DAPI (blue). Scale bar 10 μm (merge), inset scale: 4x the magnification of the merge image. **B**. Quantification of **A** as percentage of lysosomes filled with HaloTag. Images analyzed with LysoQuant. Statistical analysis: One-way ANOVA followed by Dunnett’s multiple comparison test, ^ns^P>0.05, ****P<0.0001. n = 13, 10, 13, 9 and 8 cells, respectively; n = 1 biological replicate, Mean bar is shown. **C.** Quantification of **A** as absolute number of lysosomes filled with HaloTag per cell. Images analyzed with LysoQuant. Statistical analysis: One-way ANOVA followed by Dunnett’s multiple comparison test, ^ns^P>0.05, ***P<0.001, ****P<0.0001. Mean bar is shown. **D.** Top panel: generation of the 33kDa Halo fragment in HEK293 cells expressing β-Gal-HaloTag variants or empty pcDNA3.1 vector and incubated with 100nM TMR and 50nM BafA1 or DMSO for 15 h. Middle panel: corresponding IB analysis with antibody against HaloTag showing overexpressed β-Gal-HaloTag (121kDa) and lysosomal Halo fragment (33kDa). Bottom panel: corresponding Coomassie stain as a loading control. **E**. Quantification of the formed Halo fragment 33kDa HaloTag fragment in **D**. Fragment generated in cells expressing β-Gal-HaloTag is set at 100%, normalized to Coomassie stain; N = 5 biological replicates for WT, p.Arg201Cys and p.Trp273Leuvariants, N = 3 for p.Ile51Thr, p.Arg59His variants. One-way ANOVA followed by Dunnett’s multiple comparison test, ^ns^P>0.05, **P<0.01. Mean bar is shown. Abbreviations: β-Gal, beta-galactosidase; DAPI, 4′,6-diamidino-2-phenylindole; IF, immunofluorescence; MEF, mouse embryonic fibroblasts; IB, immunoblot; EL, endolysosomes; HEK, human embryonic kidney; BafA1, Bafilomycin A1; TMR, tetramethylrhodamine; WT, wild type.

The same experiments were performed upon transfection of MEF cells with the HaloTag-tagged versions of WT, p.Ile51Thr, p.Arg59His, p.Arg201Cys and p.Trp273Leu β-Gal (β-Gal-HaloTag, Fig 5A–5C). The cell culture media were supplemented with 100nM of the HaloTag ligand tetramethylrhodamine (TMR) to label the β-Gal-HaloTag variants fluorescently and with 50nM BafA1 to prevent degradation of the β-Gal variants eventually reaching the lysosomal compartments. After 15 h, cells were processed for CLSM as described in the Methods section and immunostained with LAMP1 antibody (green, Fig 5A). Analyses of the micrographs (Fig 5A) and unbiased quantifications with LysoQuant (Fig 5B and 5C) mirror the results obtained with the HA-tagged versions of β-Gal, except for the p.Trp273Leu mutation, which is consistent with published data showing that p.Trp273Leu substitution produces stable, lysosome-located but catalytically altered β-Gal mutant [16, 21]. Similarly, the disease-causing mutations in the β-Gal sequence substantially reduce the percentage (Fig 5B) and the number (Fig 5C) of cellular LAMP1-positive lysosomes containing the ectopically expressed, fluorescent, HaloTag-tagged version of the polypeptides. These results confirm that modification with HaloTag does not change the intracellular fate of β-Gal WT or the four variants.

### Validation of the biochemical HaloTag cleavage assay to monitor delivery of β-Gal variants to lysosomes

The delivery of HaloTag-tagged polypeptides to the lysosomal lumen is followed by the proteolytic cleavage between the HaloTag and the tagged protein. This cleavage generates a HaloTag fragment resistant to the acidic/hydrolytic lysosomal environment when covalently modified with a fluorescent ligand [32, 33, 36]. To monitor the delivery of β-Gal variants to lysosomes, human embryonic kidney 293 (HEK293) cells were transiently transfected with plasmids encoding the β-Gal-HaloTag WT and four variants. As detailed in the Methods section, the fluorescent HaloTag ligand TMR was added to the cell culture media at a concentration of 100nM and the incubation was prolonged in the presence of 50nM BafA1, to prevent cleavage of the polypeptide delivered within the lysosomal compartments or of DMSO, to allow possible processing to proceed. Cells were detergent-solubilized and post-nuclear supernatants (PNS) were separated by SDS-PAGE. TMR-labeled polypeptides were directly visualized (in gel) with a 532-nm wavelength laser (Fig 5D, upper panel). The PNS of cells expressing WT β-Gal-HaloTag contains two fluorescent polypeptides (Fig 5D, upper panel, lane 2). The upper polypeptide band corresponds to the 121kDa, full-length WT β-Gal-HaloTag. The lower band is the 33kDa HaloTag fragment cleaved on the delivery of the WT β-Gal-HaloTag chimera within the lysosomes [32, 33, 36]. Consistently, the generation of the HaloTag fluorescent polypeptide is blocked upon lysosome inactivation in cells treated with BafA1 (Fig 5D, upper panel, lane 1). The identity of the polypeptides is confirmed by western blot with an anti-HaloTag antibody (Fig 5D, middle panel, lane 2). Confirming the CLSM images (Fig 5A–5C), the fluorescent 33kDa HaloTag fragment showing transport of the associated polypeptide to the lysosomal compartment is also generated in cells expressing the p.Trp273Leu mutant (Fig 5D, lane 6, and 5E) but not, or only in a negligible amount, in cells expressing the p.Ile51Thr, p.Arg59His, and p.Arg201Cys β-Gal-HaloTag variants (Fig 5D, lane 3–5, and 5E).

### Validation of the therapeutic potential of STARs with the HaloTag cleavage assay

**Fig 6:**
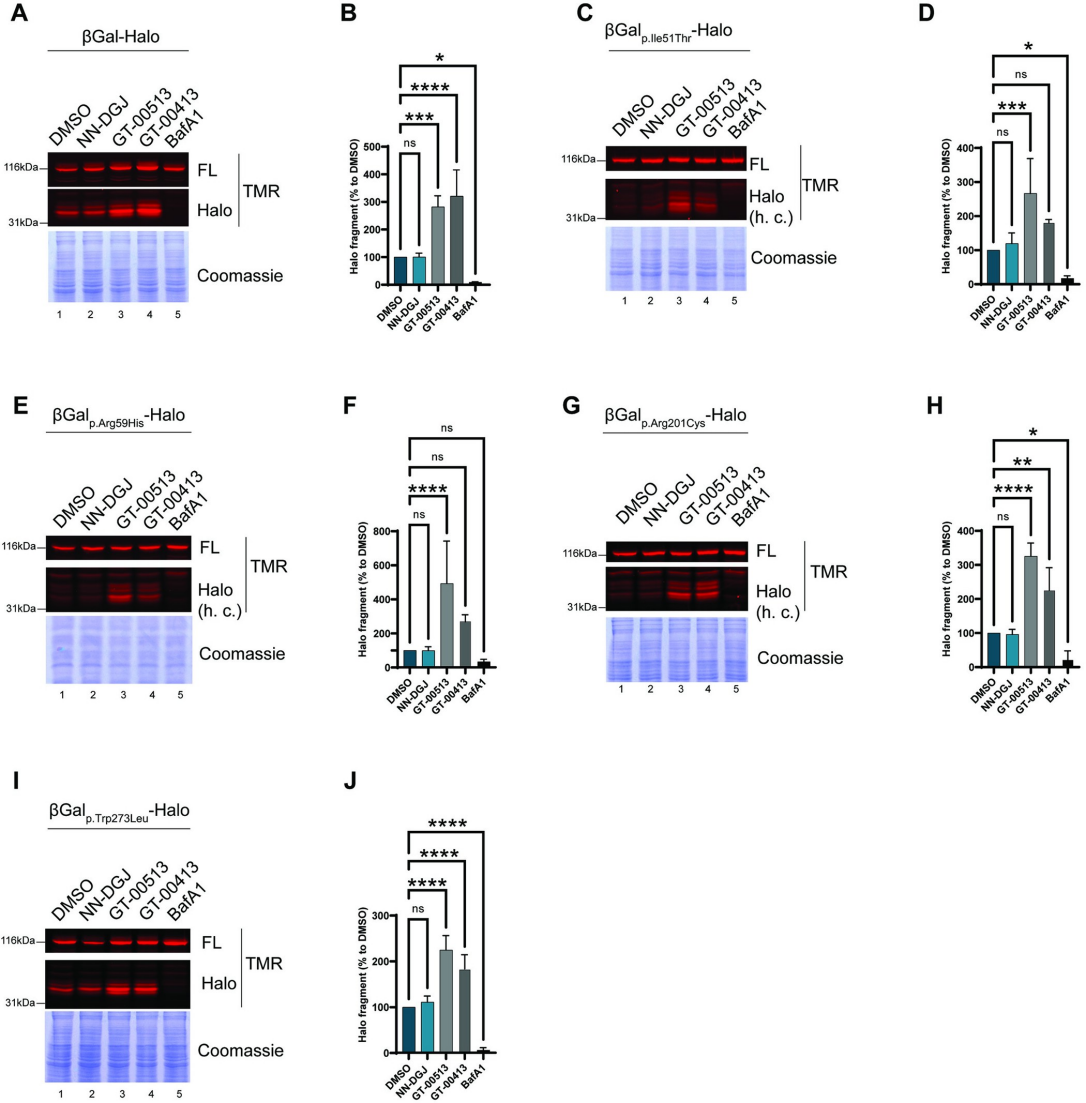
HaloTag fragment assay to measure effect of candidate allosteric pharmacological chaperones on lysosomal delivery of WT and disease-linked β-Gal-HaloTag mutants. **A.** TMR fluorescence of β-Gal-HaloTag expressed in HEK293 cells treated for four days with DMSO or GT compounds at 25 mM and incubated with 100nM TMR for the last 15 h. Control cells were treated with 50nM BafA1 only for the last 15 h. Top panel: TMR fluorescence of a band corresponding to the β-Gal-HaloTag. Middle panel: TMR fluorescence of a band corresponding to the Halo fragment. Bottom panel: corresponding Coomassie stain used as a loading control. **B**. Quantification of total Halo fragment (corrected for Coomassie signal and normalized to DMSO). Halo fragment statistical analysis: one-way ANOVA followed by Dunnett’s multiple comparison test, ^ns^P>0.05, *P<0.05, **P<0.01, ***P<0.001, ****P<0.0001; n = 3 (GT-00413) or n = 4 (GT-00513) independent experiments. Error bars show mean ± SD. **C** and **D** Same as **A** and **B** for β-Gal_p.Ile51Thr_-HaloTag; n = 3 (GT-00413) or n = 4 (GT-00513) independent experiments. **E** and **F** Same as **A** and **B** for β-Gal_p.Arg56His_-HaloTag; n = 3 (GT-00413) or n = 4 (GT-00513) independent experiments. **G** and **H** Same as **A** and **B** for β-Gal_p.Arg201Cys_-HaloTag; n = 3 (GT-00413) or n = 4 (GT-00513) independent experiments. **I** and **J** Same as **A** and **B** for β-Gal_p.Trp273Leu_-HaloTag; n = 3 (GT-00413) or n = 4 (GT-00513) independent experiments. Abbreviations: β-Gal, beta-galactosidase; DMSO, dimethylsulfoxide; IF, immunofluorescence; MEF, mouse embryonic fibroblasts; HEK, human embryonic kidney; FL, full length; h. c., high contrast; BafA1, Bafilomycin A1; NN-DGJ, N-nonyl-deoxygalactonojirimycin; SD, standard deviation; TMR, tetramethylrhodamine; WT, wild type.

Having established that the HaloTag does not affect the lysosomal delivery of β-Gal variants, we took advantage of the sensitive HaloTag cleavage assay to assess the capacity of STARs to enhance the lysosomal transport of β-Gal variants with that of the competitive inhibitor NN-DGJ. HEK293 cells were transfected with plasmids encoding β-Gal-HaloTag variants and were treated for four days with NN-DGJ or STARs (GT-00513, GT-00413) at 25 μM. Cell culture media were supplemented with 100nM of TMR ligand and the experiments were performed as described for Fig 5D. Separation of proteins contained in the PNS in SDS-PAGE and analyses of the fluorescent polypeptides confirmed the delivery of WT and p.Trp273Leu β-Gal to the lysosomes as demonstrated by the generation of the 33kDa fluorescent HaloTag fragment (Fig 6A and 6I, lane 1). As expected, lysosomal inactivation upon cell exposure to BafA1 prevented formation of these fluorescent fragments (Fig 6A and 6I, lane 5). The competitive pharmacological chaperone NN-DGJ had negligible capacity to enhance lysosomal transport of the β-Gal variants examined in this study (Fig 6A, 6C, 6E, 6G and 6I, lanes 2, with quantification in Fig 6B, 6D, 6F, 6H and 6J). In contrast, the two STAR compounds, GT-00513 and GT-00413, substantially enhanced lysosomal delivery of the WT protein (Fig 6A, lanes 3 and 4, and 6B), and the four disease-causing β-Gal variants analyzed (Fig 6C–6J). In all cases, lysosomal inactivation abolished formation of the 33kDa fluorescent HaloTag fragment (lanes 5 in Fig 6A, 6C, 6E, 6G and 6I with quantification in Fig 6B, 6D, 6F, 6H and 6J).

### STARs reduce the accumulation of toxic substrate in GM1 gangliosidosis fibroblasts

**Fig 7:**
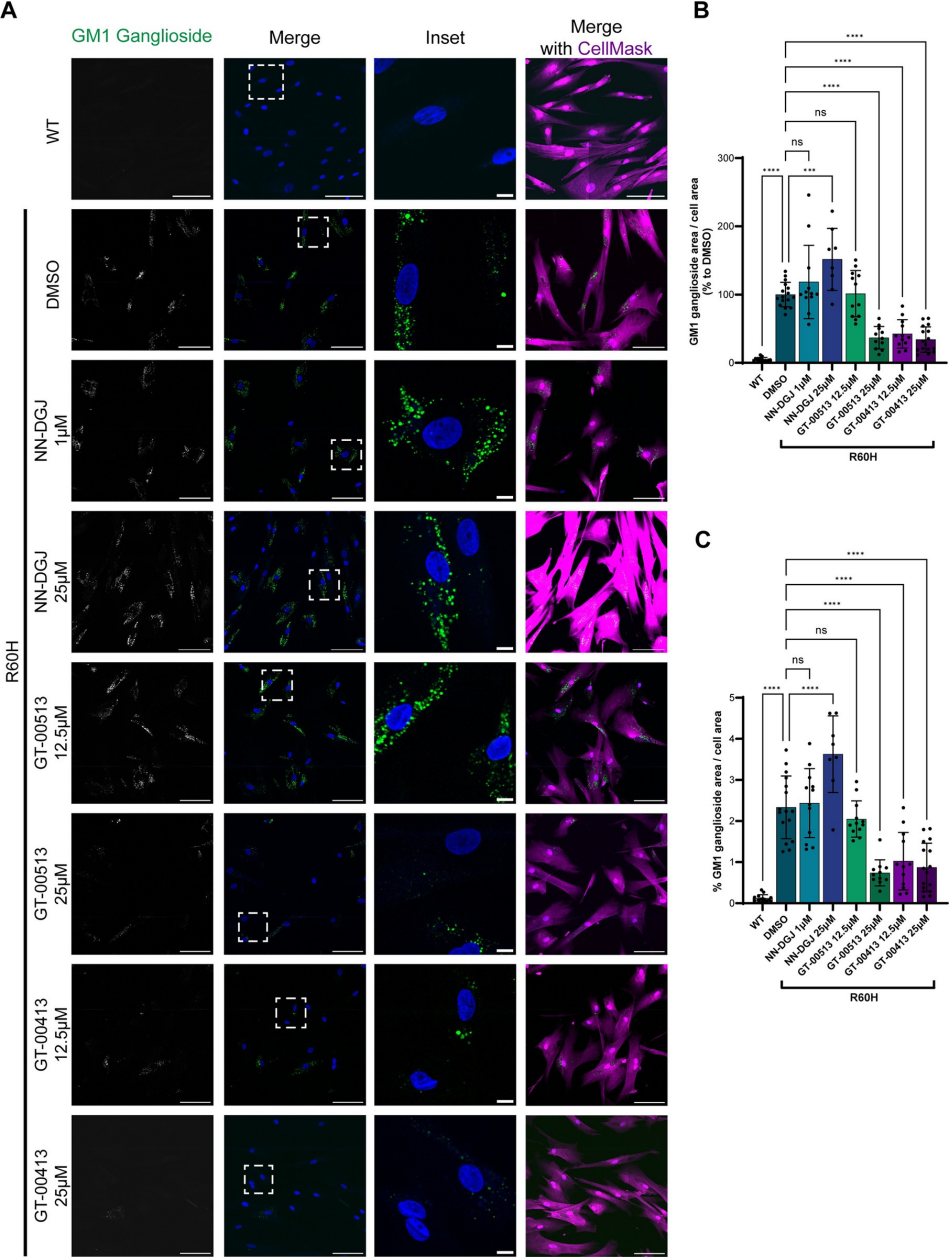
GT-00513 and GT-00413 at 25 μM reduce GM1 ganglioside accumulation in canine fibroblasts. **A**. Representative images of WT or p.Arg60His β-Gal canine fibroblasts either with DMSO or treated with indicated compounds. First column, GM1 ganglioside antibody staining (white); second column, merge of GM1 ganglioside staining (green) and DAPI (nuclei staining, blue); third column, inset of a typical region (GM1 ganglioside, green; DAPI, blue); fourth column, merge of all three channels (including CellMask, magenta). Scale bar: 100 μm, inset scale 10 μm. **B.** Quantification of GM1 ganglioside area per cell area as percentage to DMSO. **C.** Quantification of the percentage of GM1 ganglioside for the total cell area. Bars show mean±SD; n = 3 biological replicates. Significance is denoted: ^ns^P>0.05, ****P<0.0001. Abbreviations: DAPI, 4′,6-diamidino-2-phenylindole; DMSO, dimethylsulfoxide; NN-DGJ, N-nonyl-deoxygalactonojirimycin; SD, standard deviation; WT, wild type.

The ability of STARs to reduce toxic substrate was assessed in a canine fibroblast cell model bearing the missense mutation p.Arg60His. This naturally occurring p.Arg60His mutation in canine β-Gal is equivalent to the p.Arg59His prevalent mutation in human β-Gal [52]. The cultured fibroblasts were supplemented with exogenous GM1 ganglioside, whose intracellular accumulation was monitored by immunocytochemistry using specific antibodies (Fig 7). As expected, WT canine fibroblasts did not accumulate GM1 ganglioside because they have normal β-Gal function. In contrast, canine fibroblasts expressing the p.Arg60His mutant form of β-Gal showed high levels of intracellular GM1 ganglioside (Fig 7A–7C). GM1 gangliosidosis canine fibroblasts were treated for four days with NN-DGJ (as a reference control) or putative STARs GT-00513 and GT-00413. The competitive chaperone NN-DGJ had no effect in reducing exogenous GM1 ganglioside at 25 μM nor at a lower dose (1 μM) selected to avoid the inhibitory effect (Fig 7B and 7C). In contrast, both GT-00513 and GT-00413 reduced the accumulation of exogenous GM1 ganglioside. GT-00413 proved beneficial in reducing the intracellular accumulation of GM1 ganglioside at 12.5 and 25 μM, whereas GT-00513 was active at a higher dosage (25 μM), suggesting that both compounds enable mutant β-Gal to reduce the exogenous substrate, GM1 ganglioside (Fig 7B and 7C).

## Discussion

The present article used the innovative SEE-Tx® drug discovery platform to identify a new allosteric druggable binding site and discover STARs of β-Gal. Two STAR compounds, GT-00513 and GT-00413, were identified as belonging to a novel class of small molecules that bind to β-Gal and were selected for their promising drug-like profile. We assessed the mechanism of action of both STARs using a sensitive, quantitative, versatile assay to monitor lysosomal delivery of HaloTag versions of wildtype (WT) β-Gal and four disease-related β-Gal variants p.Ile51Thr, p.Arg59His, and p.Arg201Cys p.Arg60His. In this study, both STAR compounds promoted lysosomal delivery and, importantly, reduced toxic accumulation of β-Gal substrates, a hallmark of GM1 gangliosidosis. This study also confirms the applicability of SEE-Tx® to identify novel STAR compounds with therapeutic potential, as validated by the novel HaloTag-based assay.

The HaloTag cleavage assay that we developed efficiently assessed the capacity of selected STAR compounds to act as pharmacological chaperones, i.e., to enhance or restore lysosomal transport of lysosomal enzymes whose defective intracellular trafficking results in devastating LSDs [35]. Lysosomes are the site of activity for many hydrolases required for the degradation of macromolecules and maintenance of cellular homeostasis [1]. These hydrolases are synthesized in the ER, where they are folded into their correct functional conformation before entering the secretory pathway [1]. In some LSD cases, e.g., for mutations in the GLB1 gene reviewed herein, mutations in genes encoding for lysosomal enzymes cause the protein to misfold [4]. Consequently, the mutant enzyme is not able to reach the lysosomal compartment despite the active site of the enzyme remaining unaffected, which often results in a loss-of-function disease. A rational approach for pharmacological treatment is to favor the correct conformation of the mutant enzyme and promote its transport to lysosomes with the help of chaperones [53–55]. We propose that the effect of such pharmacological chaperone therapy depends on the ability of the mutant lysosomal enzymes to reach the lysosomal compartment. Therefore, to prove our hypothesis that LSD may be induced by retention of a misfolded enzymatically-intact protein within the ER, we used the HaloTag protein-tagging system [35] to develop a versatile quantitative HaloTag-based assay to characterize and monitor the delivery of mutant β-Gal to the lysosomal compartment and to assess the effect of competitive and novel allosteric chaperones.

We previously demonstrated the utility of the low pH-sensitive HaloTag-green fluorescent protein (GFP) reporter in two independent ER-phagy systems [33], which showed that accumulation of ER-resident Sec62 and alpha1-antitrypsin (Glu342Lys) Z variant (ATZ) proteins within active endolysosomes could be distinguished by the appearance of single-positive fluorescent species using confocal analysis, protease-resistant HaloTag fragments using biochemical assays, and ratiometric changes in flow cytometry profiles of the HaloTag-GFP reporter-expressing cells [32, 33]. The versatility of HaloTag-based assays for investigating ER-to-lysosome transport was further expanded by the abundance of cell-permeable fluorescent and functional ligands, allowing diverse quantitative (and time-resolved) fluorescent, electron microscopy, flow cytometry and biochemical setups [32, 33]. In this study, we used the single HaloTag in CLSM and biochemistry assays to characterize the delivery pattern of the lysosomal enzyme β-Gal and four of its misfolded, mutant counterparts. Importantly, modifications of WT and variant β-Gal with the HaloTag did not affect delivery to lysosomes, suggesting that this approach can be robustly applied to characterize lysosomal transport of disease-causing mutant, misfolded lysosomal enzymes [55]. Furthermore, the increase of lysosomal Halo fragments observed upon treatment of cells expressing mutant β-Gal with allosteric GT chaperones serves as proof-of-concept for the use of HaloTag-based assays for the identification of therapeutically active chaperones to treat LSDs. The increase in the lysosomal transport led to an elevation of enzyme activity at the site of action, effectively preventing the toxic accumulation of intracellular enzyme substrate [56, 57]. Finally, we propose to employ a synergistic screening approach combining HaloTag lysosomal trafficking assays with substrate accumulation assays to identify pharmacological compounds that promote the lysosomal delivery and functional activity of mutant enzymes.

## Conclusions

We identified two structurally targeted allosteric regulators of β-Gal (GT-00413 and GT-00513) that restored key biological activities of β-Gal found to be impaired in GLB1-related disorders. Further preclinical development of these two STARs is therefore warranted. Our results support and validate the application of the proprietary SEE-Tx® platform as a fast and cost-effective innovative drug discovery approach for identifying STARs for the treatment of GLB1-related disorders. Furthermore, our results provide proof of concept that putative STARs identified via the SEE-TX® platform can be characterized using HaloTag-based assays and potentially enter the drug development pipeline to treat LSDs. Automation and upscaling of the HaloTag-based assays proposed in this study could serve as a basis for a drug discovery platform for screening chaperones promoting the transport of mutant enzymes. In addition, the use of HaloTag ligands should not be limited to studies of ER-to-lysosome transport. It could also, theoretically, be applied to investigate the lysosomal delivery of macromolecules, cellular pathogens, and proteins located inside or on the surface of organelles or cytosolic cargo proteins.

## Supporting information

S1 Table Primers used for site directed mutagenesis. (PDF)

S2 Table Details of antibodies used. (PDF)

S1 File Enzyme inhibition assay. (DOCX)

## References

[1] Hebert DN, Molinari M. In and out of the ER: protein folding, quality control, degradation, and related human diseases. Physiol Rev. 2007;87(4):1377–408. doi: 10.1152/physrev.00050.2006 17928587

[2] Fregno I, Molinari M. Proteasomal and lysosomal clearance of faulty secretory proteins: ER-associated degradation (ERAD) and ER-to-lysosome-associated degradation (ERLAD) pathways. Crit Rev Biochem Mol Biol. 2019;54(2):153–63. doi: 10.1080/10409238.2019.1610351 31084437

[3] Sun Z, Brodsky JL. Protein quality control in the secretory pathway. J Cell Biol. 2019;218(10):3171–87. doi: 10.1083/jcb.201906047 31537714 PMC6781448

[4] Taipale M. Disruption of protein function by pathogenic mutations: common and uncommon mechanisms. Biochem Cell Biol. 2019;97(1):46–57. doi: 10.1139/bcb-2018-0007 29693415

[5] Cho SY, Jin D-K. GLB1-related disorders: GM1 gangliosidosis and Morquio B disease. J Genet Med. 2021;18(1):16–23. doi: 10.5734/JGM.2021.18.1.16

[6] Morita M, Saito S, Ikeda K, Ohno K, Sugawara K, Suzuki T, et al. Structural bases of GM1 gangliosidosis and Morquio B disease. J Hum Genet. 2009;54(9):510–5. doi: 10.1038/jhg.2009.70 19644515

[7] Yoshida K, Oshima A, Shimmoto M, Fukuhara Y, Sakuraba H, Yanagisawa N, et al. Human beta-galactosidase gene mutations in GM1-gangliosidosis: a common mutation among Japanese adult/chronic cases. Am J Hum Genet. 1991;49(2):435–42. 1907800 PMC1683306

[8] Silva CM, Severini MH, Sopelsa A, Coelho JC, Zaha A, d’Azzo A, et al. Six novel beta-galactosidase gene mutations in Brazilian patients with GM1-gangliosidosis. Hum Mutat. 1999;13(5):401–9. doi: 10.1002/(SICI)1098-1004(1999)13:5&lt;401::AID-HUMU9&gt;3.0.CO;2-N 10338095

[9] Santamaria R, Chabás A, Coll MJ, Miranda CS, Vilageliu L, Grinberg D. Twenty-one novel mutations in the GLB1 gene identified in a large group of GM1-gangliosidosis and Morquio B patients: possible common origin for the prevalent p.R59H mutation among gypsies. Hum Mutat. 2006;27(10):1060. doi: 10.1002/humu.9451 16941474

[10] Hofer D, Paul K, Fantur K, Beck M, Bürger F, Caillaud C, et al. GM1 gangliosidosis and Morquio B disease: expression analysis of missense mutations affecting the catalytic site of acid beta-galactosidase. Hum Mutat. 2009;30(8):1214–21. doi: 10.1002/humu.21031 19472408

[11] Nishimoto J, Nanba E, Inui K, Okada S, Suzuki K. GM1-gangliosidosis (genetic beta-galactosidase deficiency): identification of four mutations in different clinical phenotypes among Japanese patients. Am J Hum Genet. 1991;49(3):566–74. 1909089 PMC1683129

[12] Abumansour IS, Yuskiv N, Paschke E, Stockler-Ipsiroglu S. Morquio-B disease: Clinical and genetic characteristics of a distinct GLB1-related dysostosis multiplex. JIMD Rep. 2019;51(1):30–44. doi: 10.1002/jmd2.12065 32071837 PMC7012745

[13] Nicoli E-R, Annunziata I, d’Azzo A, Platt FM, Tifft CJ, Stepien KM. GM1 Gangliosidosis—A Mini-Review. Front Genet. 2021;12. doi: 10.3389/fgene.2021.734878 34539759 PMC8446533

[14] Suzuki H, Ohto U, Higaki K, Mena-Barragán T, Aguilar-Moncayo M, Ortiz Mellet C, et al. Structural basis of pharmacological chaperoning for human β-galactosidase. J Biol Chem. 2014;289(21):14560–8. doi: 10.1074/jbc.M113.529529 24737316 PMC4031513

[15] Caciotti A, Bardelli T, Cunningham J, D’Azzo A, Zammarchi E, Morrone A. Modulating action of the new polymorphism L436F detected in the GLB1 gene of a type-II GM1 gangliosidosis patient. Hum Genet. 2003;113(1):44–50. doi: 10.1007/s00439-003-0930-8 12644936

[16] Oshima A, Yoshida K, Shimmoto M, Fukuhara Y, Sakuraba H, Suzuki Y. Human beta-galactosidase gene mutations in morquio B disease. Am J Hum Genet. 1991;49(5):1091–3. 1928092 PMC1683264

[17] Tatano Y, Takeuchi N, Kuwahara J, Sakuraba H, Takahashi T, Takada G, et al. Elastogenesis in cultured dermal fibroblasts from patients with lysosomal beta-galactosidase, protective protein/cathepsin A and neuraminidase-1 deficiencies. J Med Invest. 2006;53(1–2):103–12. doi: 10.2152/jmi.53.103 16538002

[18] Liguori L, Monticelli M, Allocca M, Hay Mele B, Lukas J, Cubellis MV, et al. Pharmacological Chaperones: A Therapeutic Approach for Diseases Caused by Destabilizing Missense Mutations. Int J Mol Sci. 2020;21(2):489. doi: 10.3390/ijms21020489 31940970 PMC7014102

[19] Losada Díaz JC, Cepeda Del Castillo J, Rodriguez-López EA, Alméciga-Díaz CJ. Advances in the Development of Pharmacological Chaperones for the Mucopolysaccharidoses. Int J Mol Sci. 2019;21(1):232. doi: 10.3390/ijms21010232 31905715 PMC6981736

[20] Molinari M. N-glycan structure dictates extension of protein folding or onset of disposal. Nat Chem Biol. 2007;3(6):313–20. doi: 10.1038/nchembio880 17510649

[21] Fantur K, Hofer D, Schitter G, Steiner AJ, Pabst BM, Wrodnigg TM, et al. DLHex-DGJ, a novel derivative of 1-deoxygalactonojirimycin with pharmacological chaperone activity in human G(M1)-gangliosidosis fibroblasts. Mol Genet Metab. 2010;100(3):262–8. doi: 10.1016/j.ymgme.2010.03.019 20409738

[22] Steiner AJ, Schitter G, Stütz AE, Wrodnigg TM, Tarling CA, Withers SG, et al. 1-Deoxygalactonojirimycin-lysine hybrids as potent D-galactosidase inhibitors. Bioorg Med Chem. 2008;16(24):10216–20. doi: 10.1016/j.bmc.2008.10.054 18996021 PMC2910748

[23] Suzuki Y, Ogawa S, Sakakibara Y. Chaperone therapy for neuronopathic lysosomal diseases: competitive inhibitors as chemical chaperones for enhancement of mutant enzyme activities. Perspect Medicin Chem. 2009;3:7–19. doi: 10.4137/pmc.s2332 19812739 PMC2754921

[24] Valenzano KJ, Khanna R, Powe AC, Boyd R, Lee G, Flanagan JJ, et al. Identification and characterization of pharmacological chaperones to correct enzyme deficiencies in lysosomal storage disorders. Assay Drug Dev Technol. 2011;9(3):213–35. doi: 10.1089/adt.2011.0370 21612550 PMC3102255

[25] Parenti G. Treating lysosomal storage diseases with pharmacological chaperones: from concept to clinics. EMBO Mol Med. 2009;1(5):268–79. doi: 10.1002/emmm.200900036 20049730 PMC3378140

[26] Lindquist SL, Kelly JW. Chemical and biological approaches for adapting proteostasis to ameliorate protein misfolding and aggregation diseases: progress and prognosis. Cold Spring Harb Perspect Biol. 2011;3(12):a004507. doi: 10.1101/cshperspect.a004507 21900404 PMC3225948

[27] Tran ML, Génisson Y, Ballereau S, Dehoux C. Second-Generation Pharmacological Chaperones: Beyond Inhibitors. Molecules. 2020;25(14):3145. doi: 10.3390/molecules25143145 32660097 PMC7397201

[28] Fregno I, Fasana E, Bergmann TJ, Raimondi A, Loi M, Soldà T, et al. ER-to-lysosome-associated degradation of proteasome-resistant ATZ polymers occurs via receptor-mediated vesicular transport. Embo j. 2018;37(17). doi: 10.15252/embj.201899259 30076131 PMC6120659

[29] Fregno I, Fasana E, Soldà T, Galli C, Molinari M. N-glycan processing selects ERAD-resistant misfolded proteins for ER-to-lysosome-associated degradation. The EMBO Journal. 2021;40(15):e107240. doi: 10.15252/embj.2020107240 34152647 PMC8327951

[30] Fumagalli F, Noack J, Bergmann TJ, Cebollero E, Pisoni GB, Fasana E, et al. Translocon component Sec62 acts in endoplasmic reticulum turnover during stress recovery. Nat Cell Biol. 2016;18(11):1173–84. doi: 10.1038/ncb3423 27749824

[31] Loi M, Raimondi A, Morone D, Molinari M. ESCRT-III-driven piecemeal micro-ER-phagy remodels the ER during recovery from ER stress. Nature Communications. 2019;10(1):5058. doi: 10.1038/s41467-019-12991-z 31699981 PMC6838186

[32] Rudinskiy M, Bergmann TJ, Molinari M. Quantitative and time-resolved monitoring of organelle and protein delivery to the lysosome with a tandem fluorescent Halo-GFP reporter. Mol Biol Cell. 2022;33(6):ar57. doi: 10.1091/mbc.E21-10-0526 35108065 PMC9265146

[33] Rudinskiy M, Molinari M. Tandem fluorescent Halo-GFP reporter for quantitative and time-resolved monitoring of organelle and protein delivery to lysosomes. Autophagy Reports. 2022;1(1):187–91. doi: 10.1080/27694127.2022.2061679 PMC9265146 35108065

[34] Los GV, Encell LP, McDougall MG, Hartzell DD, Karassina N, Zimprich C, et al. HaloTag: a novel protein labeling technology for cell imaging and protein analysis. ACS Chem Biol. 2008;3(6):373–82. doi: 10.1021/cb800025k 18533659

[35] England CG, Luo H, Cai W. HaloTag technology: a versatile platform for biomedical applications. Bioconjug Chem. 2015;26(6):975–86. doi: 10.1021/acs.bioconjchem.5b00191 25974629 PMC4482335

[36] Yim WW, Yamamoto H, Mizushima N. A pulse-chasable reporter processing assay for mammalian autophagic flux with HaloTag. Elife. 2022;11. Epub doi: 10.7554/eLife.78923 . 35938926 PMC9385206

[37] Kimple ME, Brill AL, Pasker RL. Overview of affinity tags for protein purification. Curr Protoc Protein Sci. 2013;73(9):1–23. doi: 10.1002/0471140864.ps0909s73 24510596 PMC4527311

[38] Pérez-Carmona N, Garcia-Collazo AM, Cubero E, Bergmann TJ, Ruano A, Delgado A, et al. Insights into the mechanism of action of structurally targeted allosteric regulators for the treatment of Gaucher disease. ePoster presented at the virtual 17th Annual WORLDSymposium™ 2021.

[39] Ohto U, Usui K, Ochi T, Yuki K, Satow Y, Shimizu T. Crystal structure of human β-galactosidase: structural basis of Gm1 gangliosidosis and morquio B diseases. J Biol Chem. 2012;287(3):1801–12. Epub 2011/11/28. doi: 10.1074/jbc.M111.293795 22128166 PMC3265862

[40] Seco J, Luque FJ, Barril X. Binding site detection and druggability index from first principles. J Med Chem. 2009;52(8):2363–71. Epub 2009/03/20. doi: 10.1021/jm801385d 19296650

[41] Alvarez-Garcia D, Barril X. Molecular Simulations with Solvent Competition Quantify Water Displaceability and Provide Accurate Interaction Maps of Protein Binding Sites. Journal of Medicinal Chemistry. 2014;57(20):8530–9. doi: 10.1021/jm5010418 25275946

[42] Alvarez-Garcia D, Schmidtke P, Cubero E, Barril X. Extracting Atomic Contributions to Binding Free Energy Using Molecular Dynamics Simulations with Mixed Solvents (MDmix). Curr Drug Discov Technol. 2022;19(2):62–8. Epub 2021/12/25. doi: 10.2174/1570163819666211223162829 34951392 PMC9906626

[43] Barril Alonso X, Alvarez Garcia D, P S. Method of binding site and binding energy determination by mixed explicit solvent simulations. Patent WO2013092922A2 2012 [cited 2022 December]. Available from: https://patentimages.storage.googleapis.com/77/0b/d3/ffef95960db6fe/WO2013092922A2.pdf.

[44] Ruiz-Carmona S, Alvarez-Garcia D, Foloppe N, Garmendia-Doval AB, Juhos S, Schmidtke P, et al. rDock: A Fast, Versatile and Open Source Program for Docking Ligands to Proteins and Nucleic Acids. PLOS Computational Biology. 2014;10(4):e1003571. doi: 10.1371/journal.pcbi.1003571 24722481 PMC3983074

[45] Niesen FH, Berglund H, Vedadi M. The use of differential scanning fluorimetry to detect ligand interactions that promote protein stability. Nature Protocols. 2007;2(9):2212–21. doi: 10.1038/nprot.2007.321 17853878

[46] Aymami J, Barril X, Delgado A, Revés m, Lavilla R, Higaki K, et al. Enzyme enhancement therapy through non-competitive pharmacological chaperones. Proceedings IWBBIO; Granada 7–9 April, 2014. p. 390–5.

[47] Garcia-Collazo AM, Martinell M, Cubero E, Barril Alonso X, Rodriguez-Pascau L. Isoquinoline compounds, methods for their preparation, and their therapeutic uses thereof in conditions associated with the alteration of the activity of beta galactosidase. Patent WO2018122746A1 2017 [cited 2023 July]. Available from: https://patents.google.com/patent/WO2018122746A1/en.

[48] Schuck P. Use of surface plasmon resonance to probe the equilibrium and dynamic aspects of interactions between biological macromolecules. Annu Rev Biophys Biomol Struct. 1997;26:541–66. Epub 1997/01/01. doi: 10.1146/annurev.biophys.26.1.541 9241429

[49] Ramakrishnan NA, Drescher MJ, Sheikhali SA, Khan KM, Hatfield JS, Dickson MJ, et al. Molecular identification of an N-type Ca2+ channel in saccular hair cells. Neuroscience. 2006;139(4):1417–34. Epub 2006/04/04. doi: 10.1016/j.neuroscience.2006.01.064 16581196

[50] Morone D, Marazza A, Bergmann TJ, Molinari M. Deep learning approach for quantification of organelles and misfolded polypeptide delivery within degradative compartments. Mol Biol Cell. 2020;31(14):1512–24. Epub 2020/05/13. doi: 10.1091/mbc.E20-04-0269 32401604 PMC7359569

[51] Kucińska MK, Fedry J, Galli C, Morone D, Raimondi A, Soldà T, et al. TMX4-driven LINC complex disassembly and asymmetric autophagy of the nuclear envelope upon acute ER stress. Nature Communications. 2023;14(1):3497. doi: 10.1038/s41467-023-39172-3 37311770 PMC10264389

[52] Wang ZH, Zeng B, Shibuya H, Johnson GS, Alroy J, Pastores GM, et al. Isolation and characterization of the normal canine beta-galactosidase gene and its mutation in a dog model of GM1-gangliosidosis. J Inherit Metab Dis. 2000;23(6):593–606. Epub 2000/10/14. doi: 10.1023/a:1005630013448 11032334

[53] Mohamed FE, Al-Gazali L, Al-Jasmi F, Ali BR. Pharmaceutical Chaperones and Proteostasis Regulators in the Therapy of Lysosomal Storage Disorders: Current Perspective and Future Promises. Front Pharmacol. 2017;8:1–17. doi: 10.3389/fphar.2017.00448 28736525 PMC5500627

[54] Mohamed FE, Al Sorkhy M, Ghattas MA, Al-Gazali L, Al-Dirbashi O, Al-Jasmi F, et al. The pharmacological chaperone N-n-butyl-deoxygalactonojirimycin enhances β-galactosidase processing and activity in fibroblasts of a patient with infantile GM1-gangliosidosis. Hum Genet. 2020;139(5):657–73. doi: 10.1007/s00439-020-02153-3 32219518

[55] Marques ARA, Saftig P. Lysosomal storage disorders—challenges, concepts and avenues for therapy: beyond rare diseases. J Cell Sci. 2019;132(2):1–14. doi: 10.1242/jcs.221739 30651381

[56] Iwasaki H, Watanabe H, Iida M, Ogawa S, Tabe M, Higaki K, et al. Fibroblast screening for chaperone therapy in beta-galactosidosis. Brain & development. 2006;28 8:482–6. doi: 10.1016/j.braindev.2006.02.002 16617000

[57] Leinekugel P, Michel S, Conzelmann E, Sandhoff K. Quantitative correlation between the residual activity of beta-hexosaminidase A and arylsulfatase A and the severity of the resulting lysosomal storage disease. Hum Genet. 1992;88(5):513–23. doi: 10.1007/BF00219337 1348043

