# Supplementary material for: Validation of a highly sensitive HaloTag-based assay to evaluate the potency of a novel class of allosteric β-Galactosidase correctors
Source: PLoS One. 2023 Nov 29;18(11):e0294437. doi: 10.1371/journal.pone.0294437 (PMC10686464; doi:10.1371/journal.pone.0294437)
Supplement: S1 Table — (PDF) [file pone.0294437.s001.pdf]

S1 Table. Primers used for site directed mutagenesis.

Mutant	Forward primer (5'-3')	Reverse primers (5'-3')
β–Gal$_{p.Ile51Thr}$	agtgaatgcttcctgaggtgtagcgaaatggctgg	cagccatttcgctacacctcaggaagcattcact
β–Gal$_{p.Arg59His}$	gcattcactactcccatgtgccccgcttcta	tagaagcggggcacatgggagtagtgaatgc
β–Gal$_{p.Arg201Cys}$	gcgcttctgcaggaagcacaggtagtcaaaatcac	gtgattttgactacctgtgcttcctgcagaagcgc
β–Gal$_{p.Trp273Leu}$	cccagtgatctagcaagccagtatagaattcagaattg	caattctgaattctatactggcttgctagatcactggg

Abbreviations: β-Gal, β-galactosidase variants (p.Ile51Thr, p.Arg59His, p.Arg201Cys, p.Trp273Leu).
