# Supplementary material for: Validation of a highly sensitive HaloTag-based assay to evaluate the potency of a novel class of allosteric β-Galactosidase correctors
Source: PLoS One. 2023 Nov 29;18(11):e0294437. doi: 10.1371/journal.pone.0294437 (PMC10686464; doi:10.1371/journal.pone.0294437)
Supplement: S2 Table — (PDF) [file pone.0294437.s002.pdf]

**S2 Table. Details of antibodies used**.

| Antibody | Manufacturer | Catalog number / Clone | Dilution |
|---|---|---|---|
| Rabbit anti-HA | Sigma | H6908 | 1:100 (IF) |
| Goat anti-mouse HRP-conjugated | Southern Biotech | 1031-05 | 1:20’000 (IB) |
| Rat anti-LAMP1 | DSHB | 1D4B | 1:50 (IF) |
| Mouse anti-HaloTag® | Promega | G9211 | 1:1000 (IB) |
| Goat anti-rat Alexa647-conjugated | ThermoFisher Scientific | A-21247 | 1:300 (IF) |
| Goat anti-rabbit Alexa568-conjugated | ThermoFisher Scientific | A-11036 | 1:300 (IF) |
| HCS CellMask | ThermoFisher | T8787 | 1:5000 (IF) |
| Ganglioside GM1 | Abcam | ab23943 | 1:500 (IF) |
| Alexa Fluor® 488 Donkey anti-Rabbit IgG | Invitrogen | A21206 | 1:2000 (IF) |

Abbreviations: IF, immunofluorescence; IB, immunoblot; DSHB, Developmental Studies Hybridoma Bank; HA, influenza virus hemagglutinin; HRP, horseradish peroxidase; LAMP1, Lysosomal-associated membrane protein 1; IgG, immunoglobulin G.
