# Supplementary material for: Validation of a highly sensitive HaloTag-based assay to evaluate the potency of a novel class of allosteric β-Galactosidase correctors
Source: PLoS One. 2023 Nov 29;18(11):e0294437. doi: 10.1371/journal.pone.0294437 (PMC10686464; doi:10.1371/journal.pone.0294437)
Supplement: S1 File — (DOCX) [file pone.0294437.s003.docx]

**S1 File. Enzyme inhibition assay.**

In contrast to substrate-competitive chaperones, our compounds did not display inhibition of the β-galactosidase enzyme. To test enzyme inhibition, we compared our compounds with reference compound NN-DGJ in an inhibition experiment using both lysates from human fibroblasts expressing wild type β-galactosidase and purified human β-galactosidase protein (S1 Fig).

**i)**
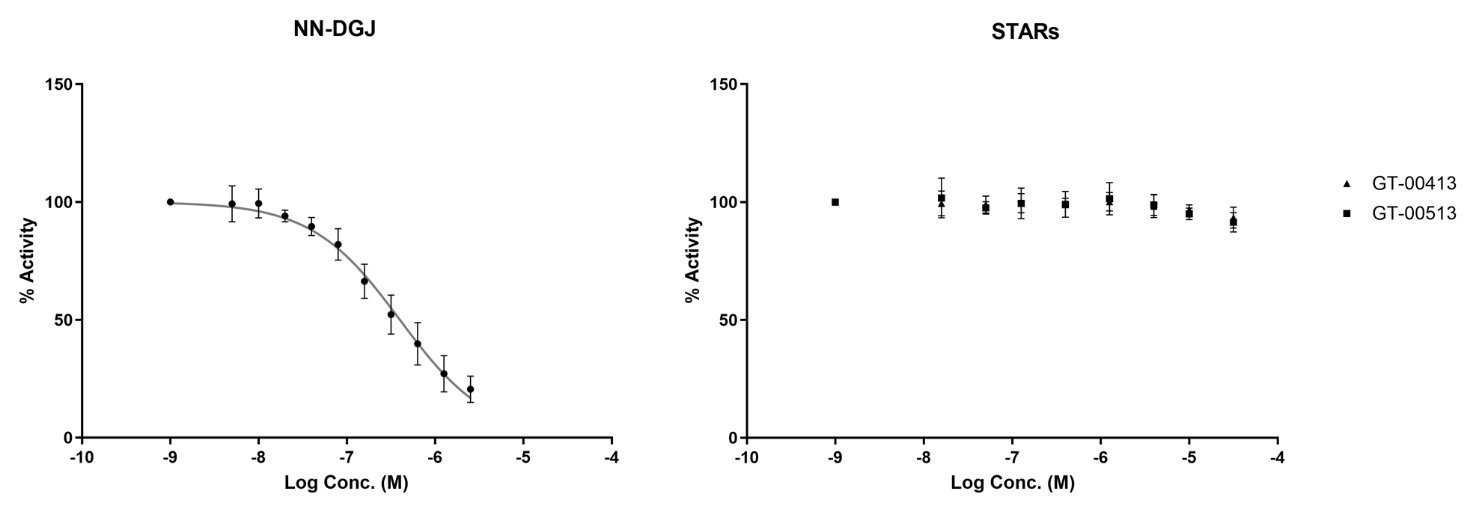


**ii)**

**
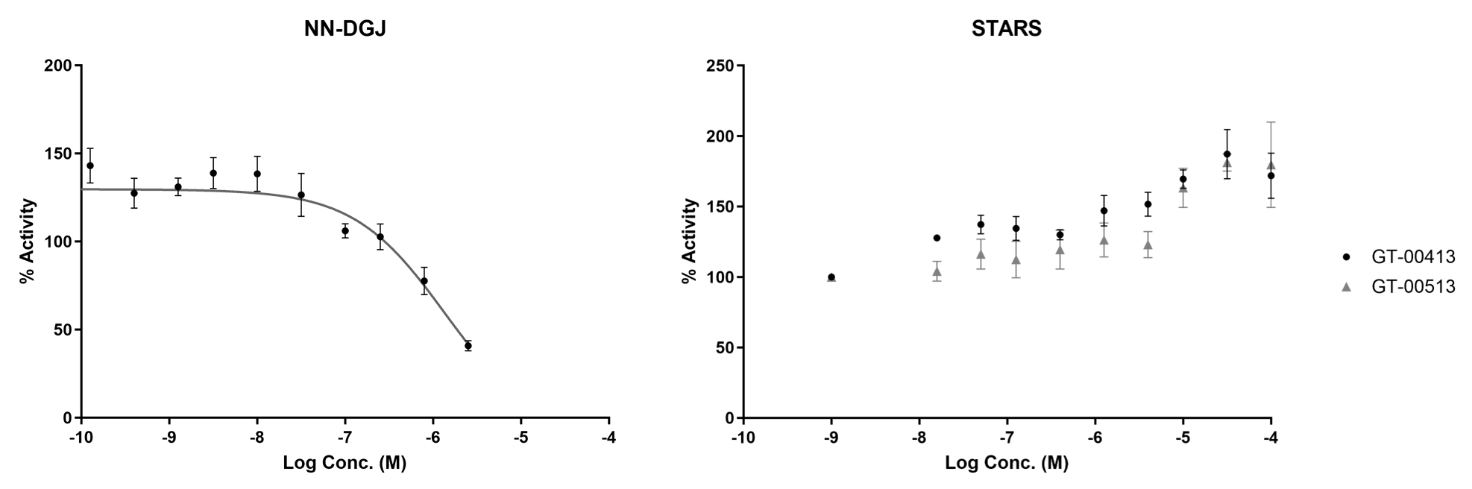
**

**A B**

S1 Fig. Inhibition of β-galactosidase in lysates from WT β-galactosidase-expressing human fibroblasts (i) and recombinant human β-galactosidase protein (ii). A) Reference compound NN-DGJ. B) Non-competitive pharmacological chaperones: GT-00513 and GT-00413 molecules

Materials and Methods

Enzyme inhibition assay.

Lysates from wild type β-galactosidase-expressing human fibroblasts

50 μL of wild-type lysate at 15 μg/mL protein concentration was diluted with 70 μL of assay buffer (50mM citrate, pH 4) and the lysate was incubated with GT compound at concentrations of 0.02–300 μM and NN-DGJ at concentrations of 0.005–2.5 μM in triplicate. Samples were then assayed for β-galactosidase activity using the resorufin β-D-galactopyranoside substrate. The assay reaction was started by the addition of 50 μL of 62.5 μM resorufin β-D-galactopyranoside in 50mM citrate buffer (pH 4) to each well. Plates were incubated at 37 °C for 2 h, and the reaction was stopped by the addition of 100 μL of glycine buffer (pH 10.7) to each well. Liberated resorufin was measured as a fluorescent signal (excitation 520nm, emission 580-640nm).

Recombinant human β-Gal protein

50 μL at 0.02 ug/mL of recombinant human β-galactosidase (Bio-Techne, Minneapolis, Minnesota, U.S.) was diluted with 70 μL of assay buffer (50mM citrate, pH 4) and the recombinant human β-Gal protein was incubated with GT compound at concentrations of 0.02–300 μM and NN-DGJ at concentrations of 0.005–2.5 μM in triplicate. Samples were then assayed for β-galactosidase activity using the resorufin β-D-galactopyranoside substrate. The assay reaction was started by the addition of 50 μL of 62.5 μM resorufin β-D-galactopyranoside in 50mM citrate buffer (pH 4) to each well. Plates were incubated at 37 °C for 2 h, and the reaction was stopped by the addition of 100 μL of glycine buffer (pH 10.7) to each well. Liberated resorufin was measured as a fluorescent signal (excitation 520nm, emission 580-640nm).
